# Review of Recent Phased Arrays for Millimeter-Wave Wireless Communication

**DOI:** 10.3390/s18103194

**Published:** 2018-09-21

**Authors:** Aqeel Hussain Naqvi, Sungjoon Lim

**Affiliations:** School of Electrical and Electronics Engineering, College of Engineering, Chung-Ang University, 221, Heukseok-Dong, Dongjak-Gu, Seoul 156-756, Korea; aqeelhnaqvi89@gmail.com

**Keywords:** beamforming, beam-scanning, millimeter-wave (mm-wave), 5G, line-of-sight (LOS), phased arrays

## Abstract

Owing to the rapid growth in wireless data traffic, millimeter-wave (mm-wave) communications have shown tremendous promise and are considered an attractive technique in fifth-generation (5G) wireless communication systems. However, to design robust communication systems, it is important to understand the channel dynamics with respect to space and time at these frequencies. Millimeter-wave signals are highly susceptible to blocking, and they have communication limitations owing to their poor signal attenuation compared with microwave signals. Therefore, by employing highly directional antennas, co-channel interference to or from other systems can be alleviated using line-of-sight (LOS) propagation. Because of the ability to shape, switch, or scan the propagating beam, phased arrays play an important role in advanced wireless communication systems. Beam-switching, beam-scanning, and multibeam arrays can be realized at mm-wave frequencies using analog or digital system architectures. This review article presents state-of-the-art phased arrays for mm-wave mobile terminals (MSs) and base stations (BSs), with an emphasis on beamforming arrays. We also discuss challenges and strategies used to address unfavorable path loss and blockage issues related to mm-wave applications, which sets future directions.

## 1. Introduction

Of the many fundamental inventions whose histories have been well documented, the origin of the antenna array is not generally known. There has been much focus on Guglielmo Marconi, who was a Nobel Prize-winning scientist, and his famous transatlantic wireless communication in December 1901 [1]. An array of 20 antenna elements was designed for the experiment. Unfortunately, strong winds destroyed the designed array system; therefore, a two-element antenna array was used to successfully transmit a repeated Morse code signal letter “S” from Poldhu, UK to St. John’s in Canada. However, another Nobel Prize-winning scientist, Luis Alvarez, was awarded the recognition globally for the discovery of the electronically scanning phased array. His innovation was initially triggered by the role of the U.S. in World War II, and was the reason for the development of *Eagle*, which was the first reported radar-based bombing system. Until now, the use of phased arrays has been very common in the defense domain, and the design of warships and military jets focus on phased array radars.

### 1.1. Phased Array

**Figure 1:**
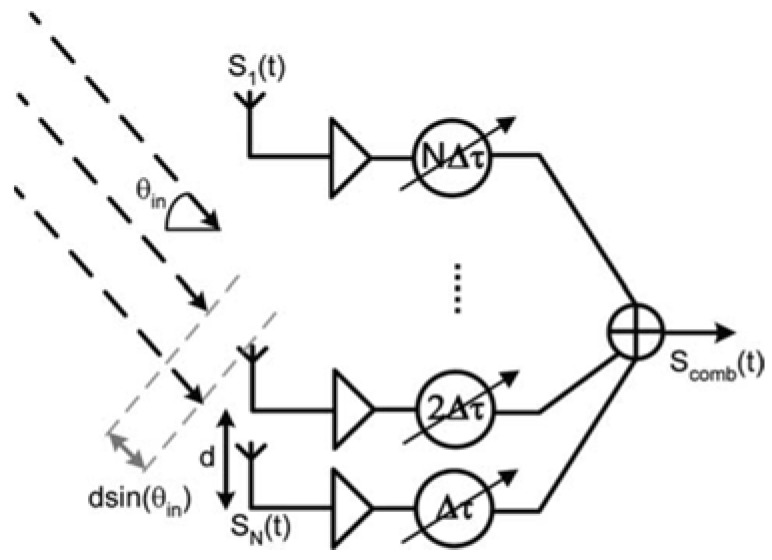
Block diagram of basic phased-array receiver [2].

A phased array is defined as a multiple-antenna system that electronically alters or directs the transmission or reception of an electromagnetic (EM) beam [1,2,3]. These systems can be realized by introducing a time variation or phase delay in every antenna’s signal path to compensate for the path differences in free space. Figure 1 shows an illustration of a phased-array receiver with N-channels. A uniform antenna spacing *d* was kept between consecutive antenna elements, and signals from various paths were combined using a variable-delay block on each signal’s path [2].

**Figure 2:**
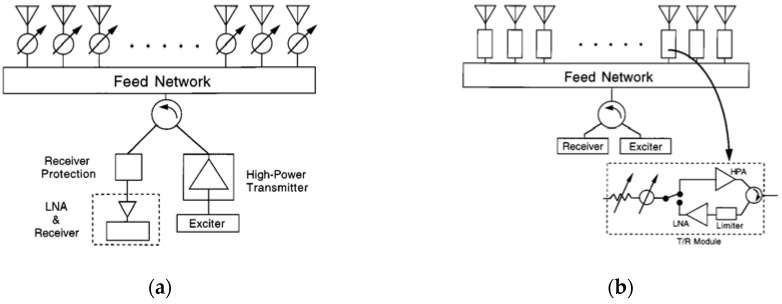
Basic architecture of (**a**) passive phased-array architecture, and (**b**) active phased-array architecture [1].

Numerous designs and structures for low-cost mm-wave electronic scanning antennas have been assessed. They contain active or passive-array structures, printed planar arrays, reflect arrays, or lens arrays. Each design may consist of different radiating elements with various properties, such as narrowband or wideband, linear or circularly polarized, digital or analog phase shifters, as well as various kinds of array feeding structures. Two fundamental phased-array structures are illustrated in Figure 2 [1].

From a radio frequency (RF) perspective, passive phased arrays differ from typical radiating arrays owing to the addition of phase shifters, which can introduce a phase delay in the array, and hence steer the beam of the antenna. Passive phased arrays do not contain active components and include a transmitter and receiver. Despite this, active phased-array structures are distributed, and contain active circuit components to produce and amplify the power. Active phased-array designs possess many benefits compared to passive-array designs, including reduced power losses and noise figures, flexible designs, and multi-function characteristics [1].

### 1.2. Millimeter-Wave 5G Wireless Communication

Over the past few decades, the continuous development of new generations of communications technology has significantly impacted the daily lives and routines of people and resulted in a constant increase in data traffic and device connections [4,5,6,7]. Different wireless services have been introduced to the market because of the rapid development of wireless communications and mobile networking techniques, which has caused smart devices to become better known, and has prompted a tremendous increase in the data traffic in wireless networks [6,7]. Owing to the increasing number of users in wireless communications, it is predicted that the number of mobile connections will surpass 100 billion by the year 2020; hence, the Internet of Things (IoT) is becoming a more common concept [5]. The use of currently unused spectra is therefore being highly encouraged because of the increasing need for higher data rates in wireless communications [4,5,6,7,8,9,10,11]. Millimeter-wave (mm-wave) communications systems have attracted much interest as a next-generation technology and are referred to as the fifth-generation (5G) wireless communication systems, which are expected to be implemented by the early 2020s [5]. The frequency spectrum specified by the International Telecommunication Union (ITU) for 5G communication includes the 3.4–3.6 GHz, 5–6 GHz, 24.25–27.5 GHz, 37–40.5 GHz, and 66–76 GHz bands, while the Federal Communications Commission (FCC) has specified the 27.5–28.35 GHz frequency band for 5G [5,6,11].

**Figure 3:**
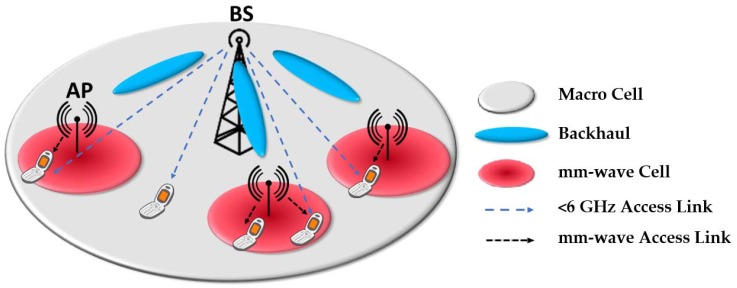
5G heterogeneous mobile network scenario.

The benefit of using mm-waves for wireless communication has been well known for a long time [4,5,6,9,10,11,12,13]. Compared with 4G, 5G wireless communication systems employ significantly different system performance scales that require data rates of the order of several gigabits-per-second (Gbps), as well as a very high data flow density, millisecond level delay, crowded connections, and enhanced spectral energy and cost factor [5,11,13,14]. It is generally agreed that the signal-to-interference-plus-noise ratio (SINR) reduces considerably owing to extreme free-space loss and blockage experienced by EM waves at high frequencies, particularly in the mm-wave bands [9]. Figure 3 illustrates a typical heterogeneous 5G mobile network scenario. The use of highly directional antennas and their line-of-sight (LOS) propagation can effectively alleviate the signal interference in between the common channels to or from other systems [14]. To achieve this, high-gain directional antennas can be used at both the transmitting and receiving ends, resulting in a significantly enhanced SINR, a reduced Doppler effect, and improved data security, and can be used in long-range mm-wave point-to-point (P2P) communications with an LOS link [6,8,10,11,13,15,16,17,18,19]. The path loss can be reduced by using directional high-gain antennas [8,9,20]. However, directional antennas with narrow beams are unsuitable for multiuser mobile communications as they provide only limited spatial coverage [6,9,20]. Moreover, directional beams need to be steered either electronically or mechanically to obtain a better substitute link for non-LOS communications [4,7,9,13,14,15,16,20,21,22,23,24,25,26,27]. Therefore, major obstacles for the implementation of commercial mm-wave systems on a large scale are their high cost and compromised performance [9,10,25].

## 2. Millimeter-Wave Antenna Array for 5G Communication

**Figure 4:**
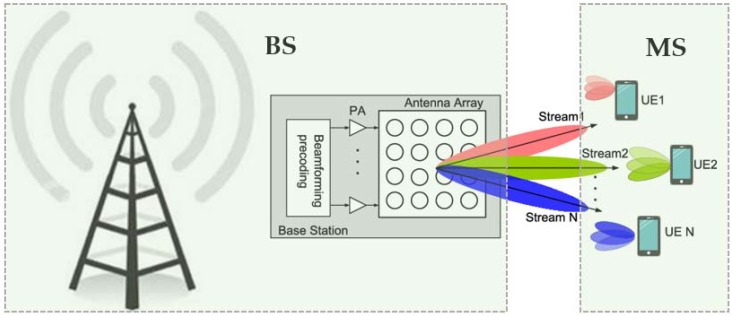
Beamforming antenna array scenario at BS and MS.

Even though mm-wave technology is generally acknowledged as being promising for 5G wireless communication systems, there exists a gap between the current mm-wave designs and the proposed commercial mm-wave cellular networks [4,5,6,9,11,13,22]. A few basic modifications are needed in the practical implementation of mm-wave cellular networks. To improve the performance of wireless communication systems, advanced antenna array architectures are used, with names such as phased arrays, beamforming arrays, and multi-input multi-output (MIMO) transceivers [1,7,9,10,12,14,15,16,17,19,22,24,25,28]. However, user mobility, the random movement of mobile devices, as well as indoor and outdoor propagation in urban environments are essential factors that affect the architecture of base station (BS) and mobile station (MS) antennas in mm-wave cellular communication systems [13,20,25,29]. Thus, to obtain a good link quality, beam-steering should be applied on both the BSs and MSs, as illustrated in Figure 4.

Generally, most of the recent mm-wave 5G research efforts are based on: (1) SiGe compound semiconductor circuits and integrated chips (ICs) [30,31,32,33,34] and (2) large-scale mm-wave phased arrays and beam-switching antennas to increase the antenna and amplifier gains within the mm-wave 5G wireless communication links [22,23]. These studies have stimulated interest in applications that focus on compact and low-power consumption, such as the mm-wave 5G backhaul, access terminals, and BSs [12,14,16]. With the help of prominent mm-wave 5G antenna studies, these applications are further specified as planar phased arrays [35], grid arrays [36], substrate-integrated waveguides (SIWs) [37], and planar lens architectures [38,39].

### 2.1. Millimeter-Wave Antenna Arrays for 5G Mobile Terminals

The application of antenna technologies for mm-wave 5G cellular handsets is still not widespread with respect to antennas architectures. Considering the significance and role of cellular phones in the mobile network industry, mm-wave antennas for 5G cellular handsets are considered as the trend-shifting technology for 5G mobile networks. The use of a mm-wave transceiver within a mobile phone presents significant challenges that should be considered [7,16]. The importance of these challenges is found because of the unique scenarios related to the use of mobile phones. Mobile phones are more compact in size relative to tablet PCs and wireless docking stations, and owing to the expanded use of smart phones, their usage scenarios are highly varied [7]. Secure and reliable communication link at Gbps speeds need to be guaranteed by the 5G transceivers within a mobile phone. Among the key factors considered in the establishment of mm-wave 5G networks, the antenna design requires the most extensive modifications. The antennas, which are technically described as omnidirectional antennas that transmit and receive RF signals equally in all horizontal directions in both geometrical planes, are the focus of resonant-type antennas implemented in user devices. The omnidirectional antennas inside mobile phones have gains that generally fall within the range of -8 to 0 dBi [28]. To compensate for the high signal attenuation at mm-wave frequencies with increased gain, the idea of antenna arrays for mobile phones was proposed.

In the wireless communication industry, the design of antennas for mobile phones is considered by RF and antenna engineers to be an engineering art. This is because of the difference in the performance of antennas when in an ideal situation in free space and when installed inside a handset. It is not possible for an antenna that is installed in a mobile phone to behave identical to one in free space without considering real-time scenarios. The induced electric and magnet field-coupling due to the antenna surface currents eventually affect the characteristic impedance matching and designed antenna efficiency in free space [16,25,40]. Moreover, the radiation performance of the antenna varies owing to the presence of other electronic components and the metallic casing [41]. Most of the space inside mobile phones is taken up by the large liquid-crystal display (LCD) and battery. The metallic brackets located behind the LCD effectively limit the design of current mobile phone antennas to the device edges [40]. Moreover, the use of certain metallic frames that are employed for different types of sensors, cameras, speakers, and microphone modules further reduce the available antenna space inside cellular phones [16]. Therefore, antenna arrays with single- and multilayer printed circuit board (PCB) technologies, polarization diversity, and a wide scanning range will be presented in this paper.

#### 2.1.1. Multilayer Phased-Array Antennas for 5G Mobile Terminals

**Figure 5:**
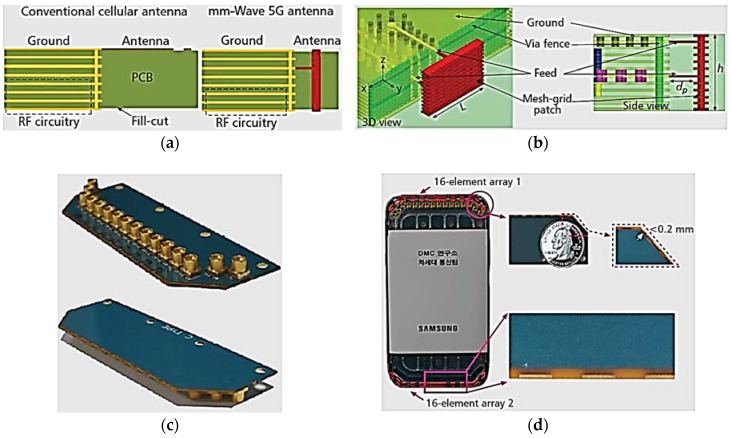

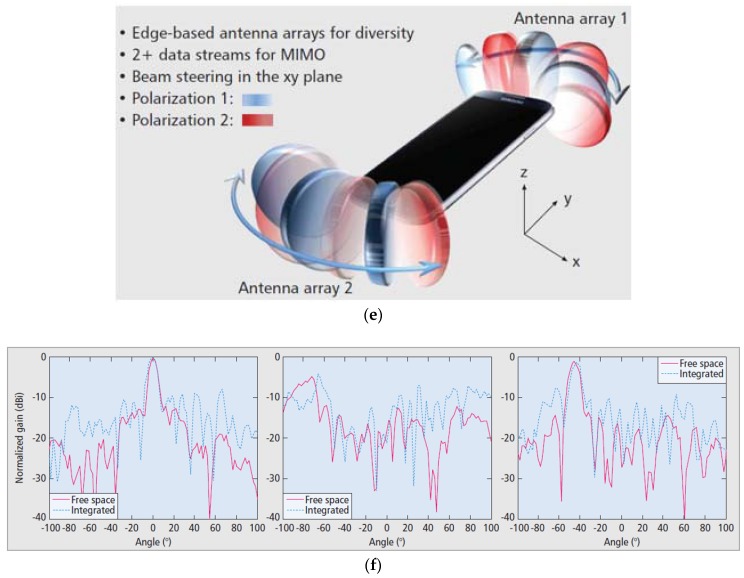
(**a**) Comparative illustration of the standard cellular antenna and mm-wave 5G antenna. (**b**) Proposed antenna. (**c**) Prototype photograph of the standalone mm-wave antenna array with coaxial connectors. (**d**) Photograph of mm-wave 5G cellular antenna array integrated inside a Samsung handset and zoomed-in views of 5G mm-wave antenna array. (**e**) mm-wave antenna array configuration for 5G cellular mobile terminals. (**f**) Measured and normalized radiation patterns for different beam directions (Figure 5f redrawn from [10]).

In [10], Hong et al. presented the basic design concept of mm-wave 5G antennas for cellular phones at 28 GHz. In that study, the design of 1 × 16 mesh-grid antenna-element phased arrays at the top and bottom positions of cellular phones has been realized using two sets of PCBs, as shown in Figure 5d. The mesh grid antenna elements are arranged in slanted angles of approximately 50° at each corner of the PCB. The proposed mesh-grid mm-wave antenna array configuration exhibits a fan-beam radiation characteristic, as shown in Figure 5e. The proposed array antenna was measured in the anechoic chamber at Samsung Electronics headquarters located in Suwon, South Korea. The measurement results show a 130° and 12° 3-dB beamwidth in the elevation and azimuth planes, respectively. The proposed array antenna exhibits a 10-dB impedance bandwidth of 1 GHz at a 27.9-GHz center frequency. The antenna array inside the cellular device has a measured peak gain of more than 10.5 dBi. The radiation pattern mismatch between the free-space and integrated scenarios is because of diffraction and refraction between the chassis and antenna elements of cellular phones. Figure 5f illustrates the radiation patterns of a mm-wave antenna array in specific beam directions for both free-space and integrated scenarios. The measurement shows an angular scanning range of ±70°. The proposed antenna array configuration on the top and bottom of the cellular device achieved an almost spherical radiation coverage, as shown in Figure 5e.

**Figure 6:**
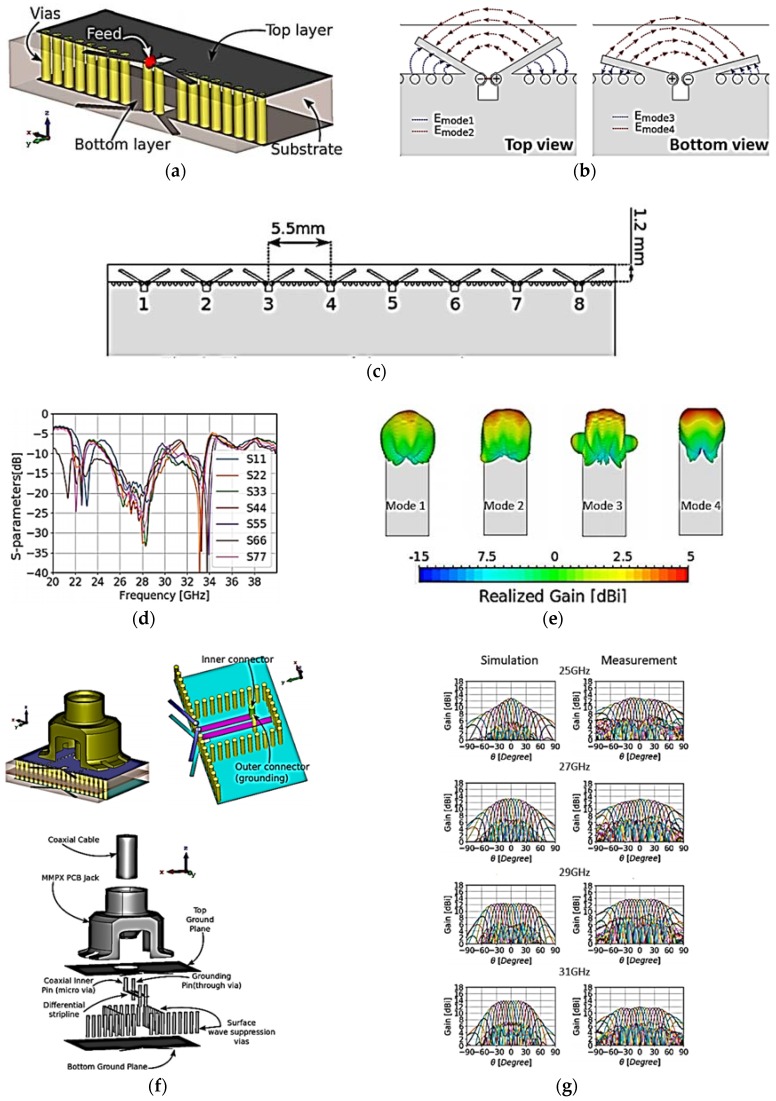

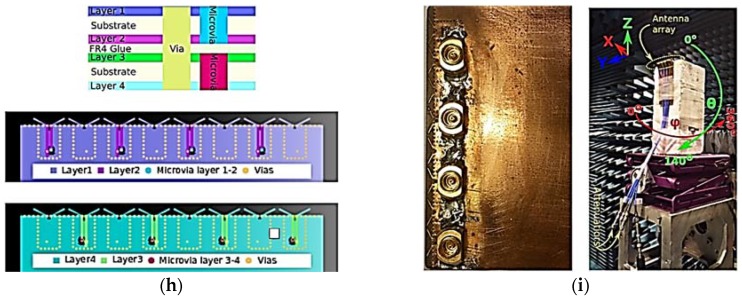
(**a**) Three-dimensional (3D) illustration of proposed antenna element. (**b**) Electric fields of proposed antenna’s modes: top view (**left**) and bottom view (**right**). (**c**) Geometry of proposed eight-element phased-array antenna. (**d**) S-parameters of proposed phased-array antenna. (**e**) 3D radiation patterns of proposed phased array in all modes. (**f**) 3D view of antenna and MMPX feeding structure. (**g**) Simulated and measured 3D radiation patterns at different scanning angles at 25, 27, 29, and 31 GHz. (**h**) Overview of the layers in the antenna structure. (**i**) Prototype of the phased-array antenna. (Redrawn from [42])

In [42], a planar quad-mode wideband phased array for a 5G mobile terminal device was reported. A phased-array design consists of eight antenna elements with eight MMPX (Micro-Miniature Connector) connectors. The proposed phased array is a multilayer design comprising two Taconic RF-30 substrate layers with thickness 0.762 mm, having a dielectric constant of 3 and loss tangent of 0.0014, vias, and microvias. Both layers were glued with FR4 glue having a thickness of 0.2 mm, and a dielectric constant of 4.3 and tangent loss of 0.025. The design overview of the proposed antenna is shown in Figure 6a. A coaxial-to-differential stripline transition feeding is proposed. The antenna is fed by a differential stripline feed. The top and bottom dipole antennas were connected to differential stripline through vias, as shown in Figure 6a. The proposed array antenna generates four distinctive modes when excited, as illustrated in Figure 6b, with an endfire radiation pattern in all modes. The measured reflection coefficients of the proposed eight-element phased-array antenna are presented in Figure 6d. The main beam-scanning range is from −90° to +90°. Figure 6g illustrates the scanning results of the proposed beam-steering phased-array antenna at 25, 27, 29, and 31 GHz. The final prototype of the phased-array antenna with connectors is shown in Figure 6i.

#### 2.1.2. Multi-Polarized Multilayer Phased-Array Antennas for 5G Mobile Terminals

To improve the diversity gain in 5G mobile terminals, the use of multi-polarized antenna arrays is a desirable solution to overcome the polarization mismatch problem. A simple demonstration dual-polarized mm-wave antenna with good isolation is reported in [43]. To achieve dual polarizations at 28 GHz, the topology of a side-by-side arrangement of horizontal and vertical Yagi-Uda antennas is used in [44]. In [45], an aperture antenna array with a dual-polarized unidirectional pattern through a back cavity on multilayer PCB technology is proposed.

**Figure 7:**
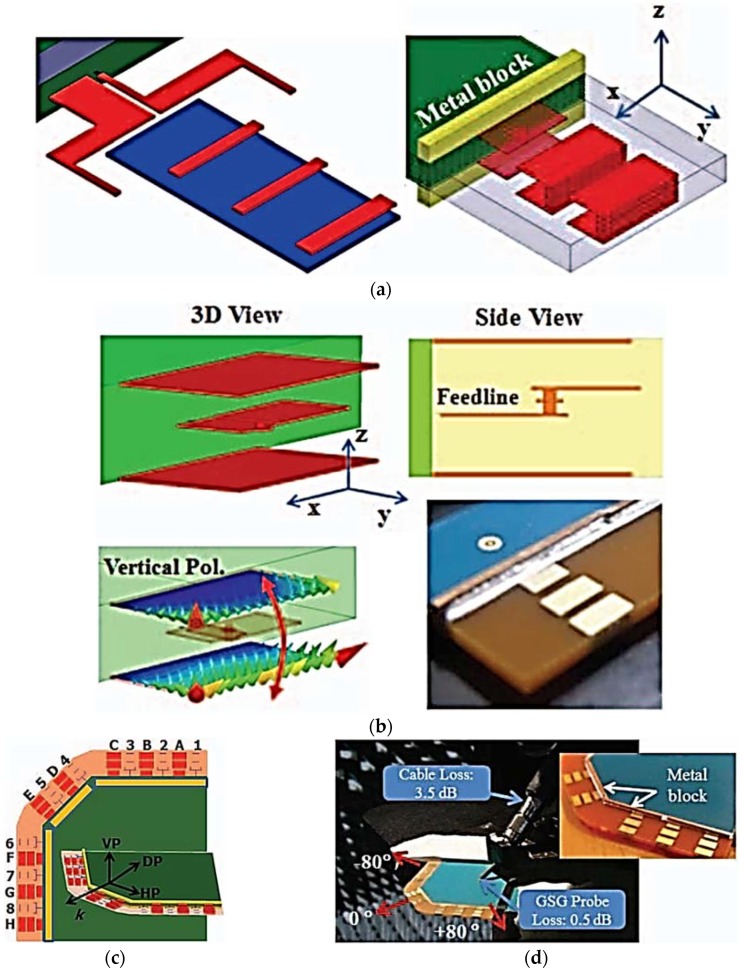
(**a**) Two discrete 28-GHz mm-wave antenna elements, i.e., the horizontally polarized planar Yagi-Uda antenna (left), and the vertically polarized multi-plate antenna (right). (**b**) Final topology of vertically polarized multi-plate antenna. (**c**) 16-element phased-array configuration at the edge of the mobile terminal. (**d**) Photograph of the prototype testing in an anechoic chamber and close-up view of 5G mm-wave phased-array antenna (Redrawn from [46]).

A multi-polarized mm-wave antenna array configuration for 5G mobile terminals was demonstrated in [46]. The proposed work is the continuation of the previously reported work of Hong et al. The reported research focuses on losses incurred by the polarization mismatch to enhance the mm-wave transmission and reception efficiency. At mm-wave frequencies, it is complicated to transmit high-power energy because of well-understood propagation and absorption losses. The 5G wireless communication link budget estimation has become more inflexible owing to real-life constraints such as limited battery life. Because mobile antennas integrated inside the mobile terminals face different angular motions, polarization mismatches between transmit (Tx) and receive (Rx) antennas have become an important loss factor for mm-wave cellular communication. To overcome the polarization mismatch loss factor, two different antenna-element designs based on the antenna array schematic are demonstrated and investigated. A coplanar waveguide-fed horizontally polarized planar Yagi-Uda antenna-element configuration together with a multi-plate antenna-element topology, which excites a vertically polarized electric field, is proposed, as shown in Figure 7a. The 16-element phased-array antenna depicted in Figure 7c is designed by deploying the two linearly polarized antenna elements alternatively along the edge of the mobile terminals with an angular scanning range of ±80°. By maintaining the distance at less than 3 mm, an isolation of more than 40 dB was achieved between the horizontally polarized and vertically polarized multi-antenna elements. The two sets of 16-element phased-array antennas on the opposite corners at the top and bottom sides of the cellular device provide maximum spherical coverage and polarization diversity. An antenna array with a maximum height of 0.8 mm was fabricated using 10-layer FR-4 lamination. The dielectric constant and tangent loss at 28 GHz are determined to be 4.2 and 0.09, respectively.

**Figure 8:**
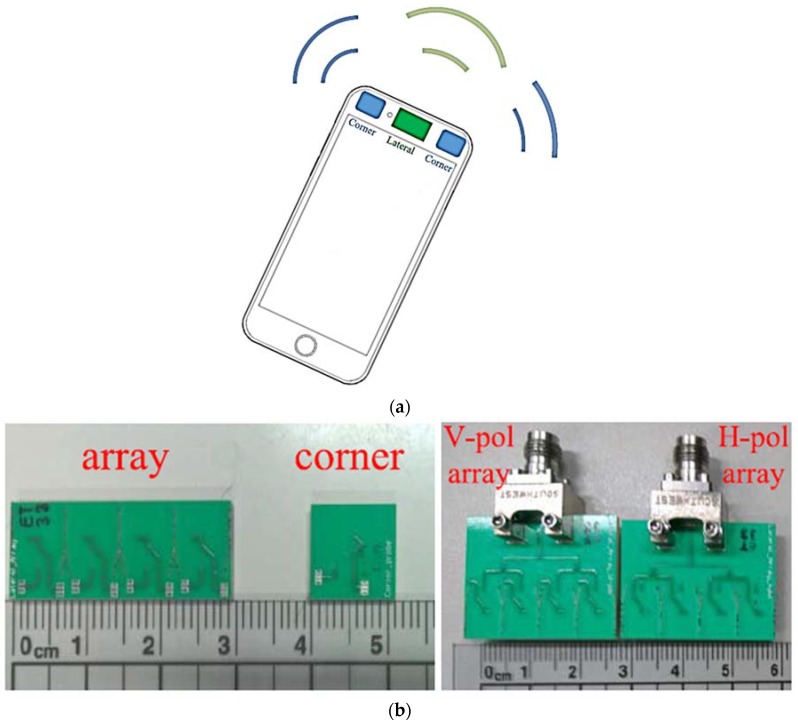
(**a**) Geometry of the mobile terminal with array position in lateral and corner edges. (**b**) Prototype photograph of the multi-polarized quasi-Yagi-Uda antenna arrays for lateral and corner edges. (Redrawn from [47])

A multi-polarized antenna array that integrates the horizontally and vertically polarized quasi-Yagi antennas together into a single area was implemented and demonstrated in [47] for 5G mobile terminals, achieving polarization diversity and size reductions. To achieve polarization diversity along with the beam-scanning capability, dual-polarized quasi-Yagi-Uda antennas for both the corner edges and a lateral array design were addressed, as illustrated in Figure 8a. The proposed array antenna was implemented on a three-layer structure on a Rogers4003 substrate with a total thickness of 1.93 mm, which has a dielectric constant of 3.38 and a tangent loss of 0.0027. The implemented horizontally polarized and vertically polarized antenna array designs for the lateral edge as well as the multi-polarized antenna for the corner edges are shown in Figure 8b. The 10-dB impedance bandwidth is 25% with a port isolation of more than 20 dB. The peak realized gain of the array exceeds 11.8 dBi, with an overall efficiency of more than 80%.

#### 2.1.3. Single-Layer Phased-Array Antennas for 5G Mobile Terminals

**Figure 9:**
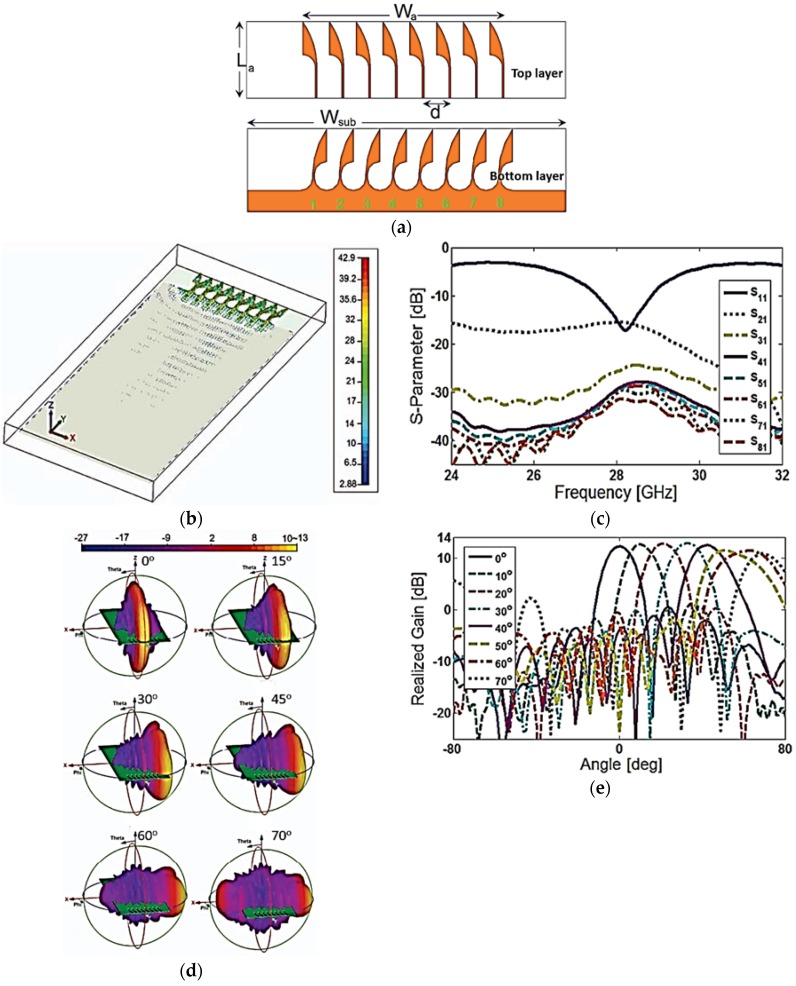
(**a**) Geometry of the Vivaldi phased-array antenna. (**b**) Surface-current distribution of proposed phased-array antenna at 28 GHz. (**c**) Simulated S-parameters of the proposed Vivaldi antenna array elements. (**d**) Radiation patterns of the antenna at different scanning angles. (**e**) Realized gain patterns of the antenna at different scanning angles. (Redrawn from [48]).

A novel design of a Vivaldi phased antenna array is presented in [48] for 5G mobile terminals. A low-cost N9000 PTFE substrate was used for the proposed phased-array antenna. The proposed array antenna comprises eight antipodal Vivaldi antenna elements. A 50-Ω microstrip feed line feeds the tapered arms on the upper side of the antenna and is used at the upper end of mobile PCB, as shown in Figure 9b. The simulated S-parameters of the eight Vivaldi elements with discrete ports are shown in Figure 9c. The antenna has an operating bandwidth of more than 1 GHz at a 28-GHz center frequency. The surface-current distribution of the proposed 5G array antenna at an operating frequency of 28 GHz is shown in Figure 9b. The proposed phased-array antenna has enough beam-scanning range in the *φ* direction ranging from 0° to 70°, as shown in Figure 9d,e.

**Figure 10:**
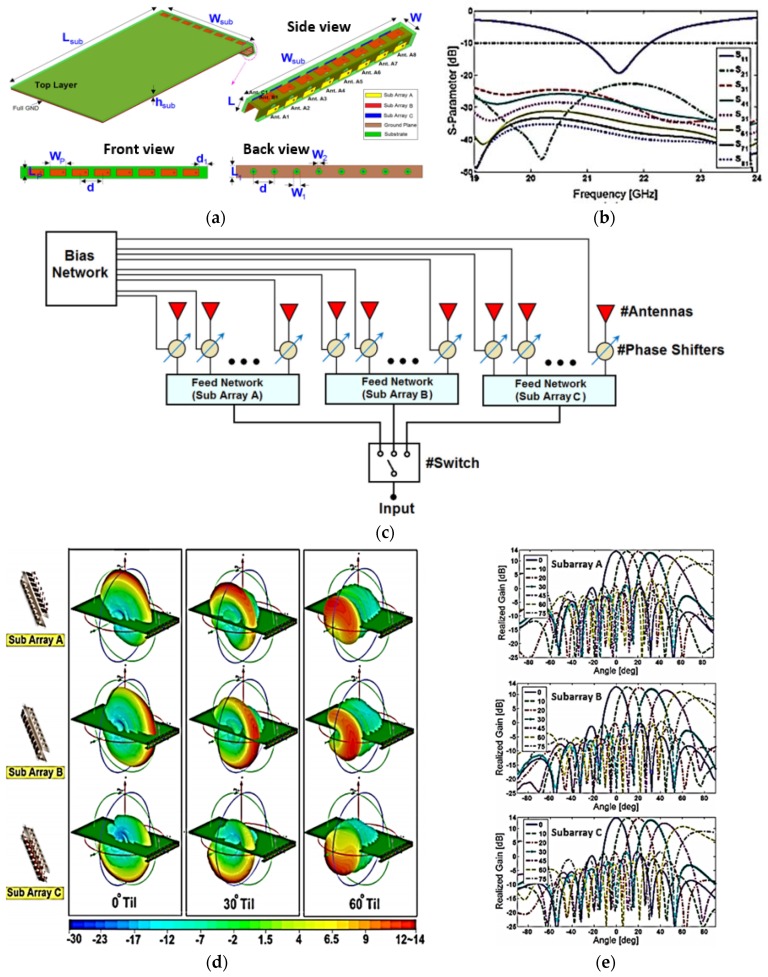
(**a**) Proposed view of 5G phased-array antenna with full ground plane. (**b**) S-parameters of proposed phased-array antenna with eight elements. (**c**) Proposed phased-array architecture. (**d**) 3D radiation patterns of each subarray at different scan angles. (**e**) 2D realized gain patterns at different scan angles. (Redrawn from [49])

A phased-array antenna with switchable three-dimensional (3D) scanning for 5G mobile terminals was proposed in [49]. Three similar subarrays of patch antennas arranged along the edge of the mobile terminal were proposed. Each subarray consists of eight microstrip patch antenna elements (MPAs) and has a beam-scanning capability of ±90° in the *θ* plane. The antenna was designed on the Nelco N9000 substrate having a thickness of 0.787 mm with a dielectric constant of 2.2 and tangent loss of 0.0009. Figure 10c illustrates an architecture to implement feeding using low-loss phase shifters with a 4.5-dB insertion loss for beam-steering, and a microwave SP3T switch to switch between the subarrays. The proposed design has a 1-GHz 10-dB impedance bandwidth in the frequency range from 21 to 22 GHz, as shown in Figure 10b. The proposed phased-array design has a good beam-scanning range of −90° to +90° with a gain of more than 12.5 dBi, as shown in Figure 10d,e.

**Figure 11:**
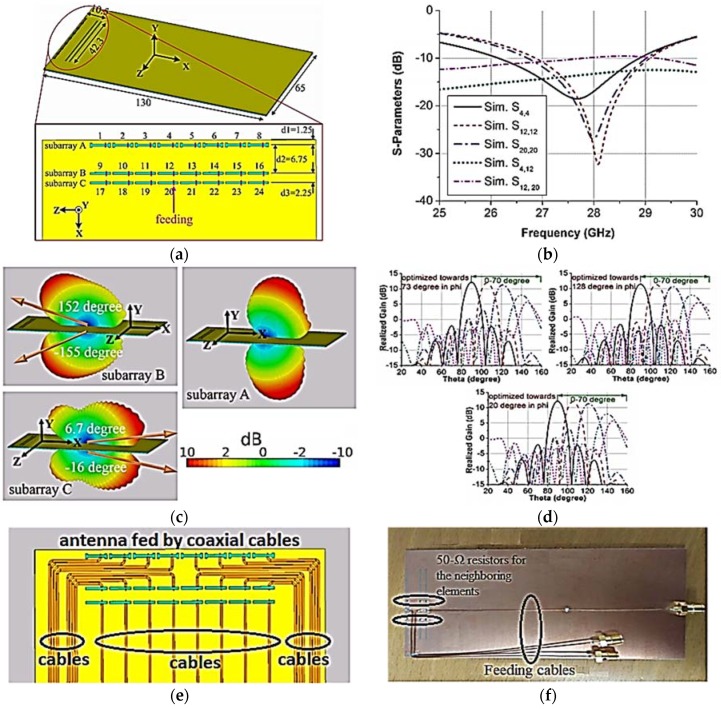
(**a**) Printed switchable array antenna configuration of subarrays. (**b**) S-parameter plots of proposed array antenna for elements 4, 12, and 20 in subarrays A, B, and C, respectively, using discrete ports. (**c**) Gain patterns with beam-steering in the *φ* direction of each subarray with all elements. (**d**) Radiation gain patterns with 70° beam-scanning: at *φ* = 73° for subarray A, at *φ* = 128° for subarray B, at *φ* = 20° for subarray C. (**e**) Illustration of proposed phased-array antenna with coaxial feed cables. (**f**) Prototype of proposed phased-array antenna with coaxial feed cables. (Redrawn from [50])

The 3D beam coverage was achieved and proposed by Zhang et al. in [50]. Three planar phased subarray configurations, as shown in Figure 11b, were used to change/switch the beam pattern to their distinct regions using chassis surface-wave excitation. The 3D spherical coverage is achieved by merging the beam patterns of subarrays. The proposed antenna has a 2-GHz 10-dB impedance bandwidth at 28 GHz, as shown in Figure 11b. A Nelco N9000 PCB substrate was used to design the antenna with a dielectric constant of 2.2 and a loss tangent of 0.0009. The substrate dimensions are 65 mm × 130 mm, with a thickness of 0.764 mm. A subarray comprises eight slot elements, each with a dimension of 4.85 mm × 0.5 mm. The consecutive slot-element distance from center-to-center is 5.35 mm. The whole phased-array switches the main beam between subarrays in the *φ* direction, and scans the beam in the *θ* direction with variable phase shifts, as shown in Figure 11c,d. All the elements between subarrays A, B and B, C have a mutual coupling lower than −12.3 dB and −9.4 dB, respectively. The angular scanning range of subarrays A, B, and C point in the *φ* directions of ±73°, ±128°, and ±20°, respectively. The efficiency of subarrays A, B, and C are 74.3%, 68%, and 72.2%, respectively.

**Figure 12:**
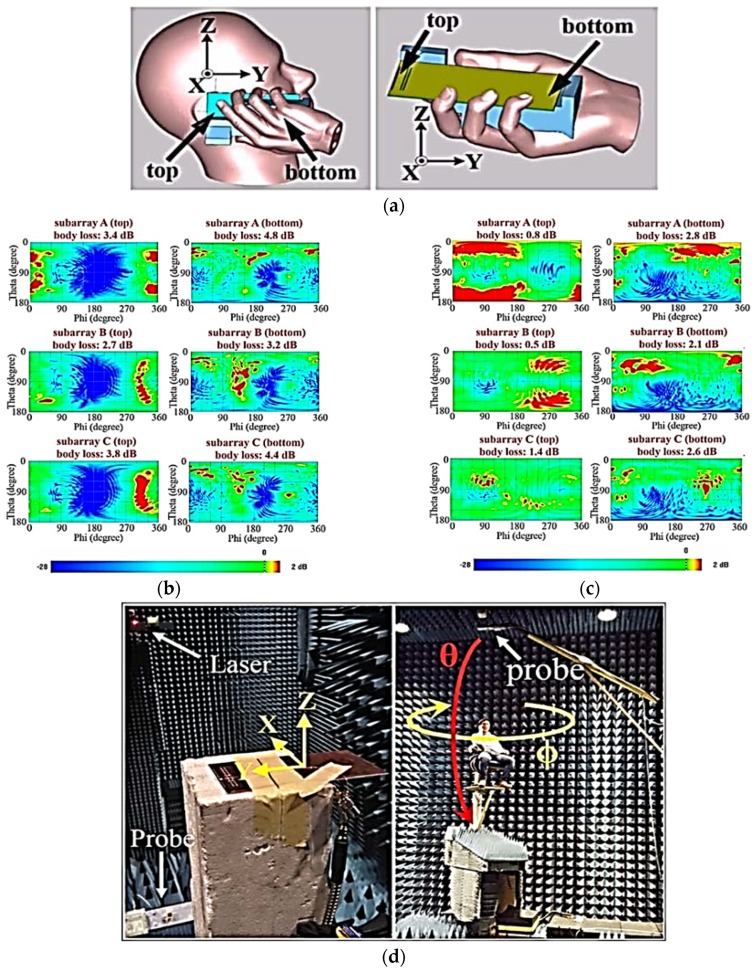
(**a**) User effect setup for: talk mode (**left**) and data mode (**right**). (**b**) Gain plots of each subarray for top and bottom sides of chassis in talking mode. (**c**) Gain plots of each subarray for top and bottom sides of chassis in data usage mode. (**d**) Measurement setup for free space (**left**) and for talking mode with real person (**right**). (Redrawn from [50])

Zhang et al. also reported a study of user effects on the proposed switchable array in user mode, i.e., talking mode and data mode, at 28 GHz. Figure 12a illustrates different setups in user mode, where ‘’top” and ‘’bottom” indicate the switchable array position at the top of the chassis near to the index finger and at the bottom of the chassis close to the hand palm, respectively. The electrical properties of skin and different tissues were taken from [50]. Parametric results for the switchable array on the top and bottom of the chassis in talking mode and data usage mode are shown in Figure 12b,c, respectively. Based on the parametric analysis and results, it is proposed that the switchable array has a better performance in terms of beam-switching, body loss, and realized gain on the chassis top position when compared with the bottom of the chassis. It is also proposed to design an additional array on the bottom side of the chassis, which results in a decreased shadowing effect in talking mode.

**Figure 13:**
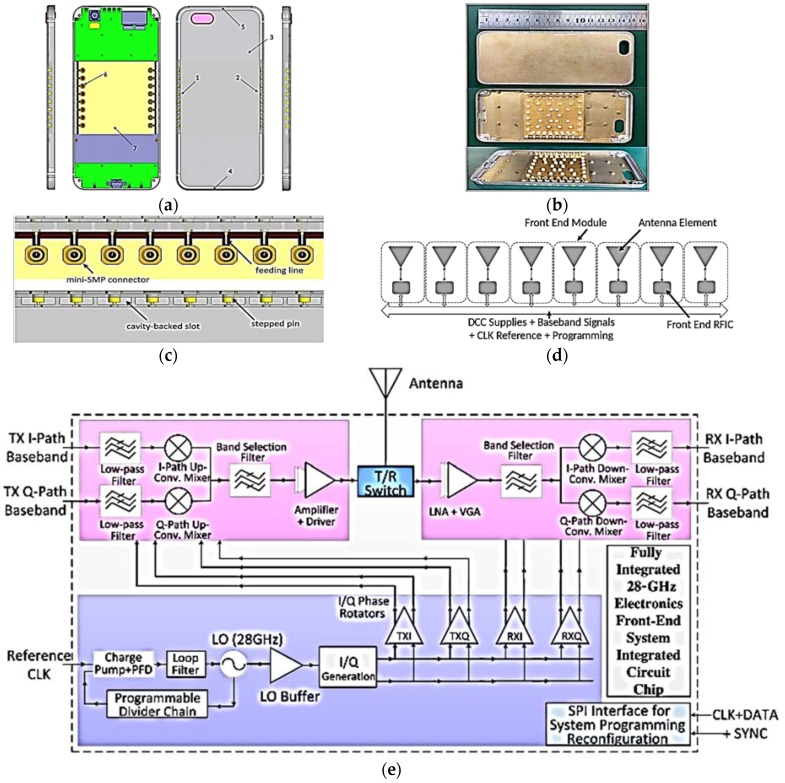

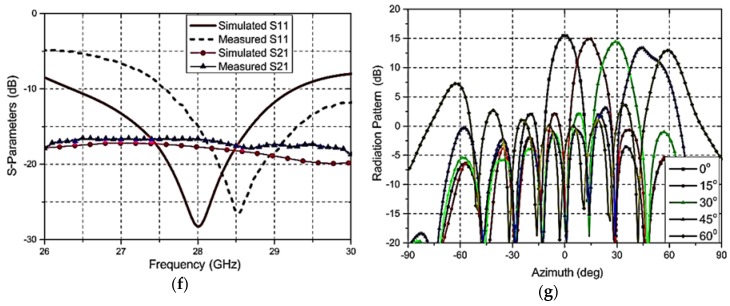
(**a**) Different views of mobile terminal design. (**b**) Photograph of mobile terminal with proposed phased-array antennas. (**c**) Design of proposed eight-element antenna array. (**d**) Block diagram of eight-element beam-steering antenna array. (**e**) Block diagram description of 28-GHz front-end RFIC chip. (**f**) Simulated and measured input reflection coefficients and isolation between array elements. (**g**) 2D radiation plots at different scanning angles (Redrawn from [41]).

Yu et al. presented a study and design of a novel phased-array antenna operating at 28 GHz with beam-steering applications for a 5G mobile terminal with metallic casing in [41]. The proposed beam-steering array comprises two subarrays, each having eight identical elements on both sides of a mobile device with a metallic casing, as shown in Figure 13a. A slot element with a cavity-backed structure is proposed, which is easy to fabricate on the metallic casing of the mobile terminal. An important factor is to determine the optimum position of the proposed phased subarrays inside the mobile terminal before finalizing the actual design in practice. The length of the slot is kept at the half-guided wavelength λ_g_/2, i.e., 5.8 mm at an operating frequency of 28 GHz, and the corresponding slot width is 1.5 mm with a cavity height of 4 mm. The high-gain directional radiation pattern of the slot element is achieved. Figure 13c illustrates the design of one of the proposed eight-element phased arrays. It is proposed to use small stepped pins soldered on the microstrip feed line feeding each element of the subarray. The microstrip feeding line is printed on a 0.254-mm-thick Rogers 5880 substrate having a dielectric constant of 2.2 and a loss tangent/of 0.0009. Each element has been provided by the phase variation using 6-bit phase shifters within a 28-GHz front-end RF integrated circuit (RFIC) chip to accomplish beam-steering, as illustrated in Figure 13e. Figure 13d shows the block diagram of the eight-element beam-steering phased array.

Figure 13f shows the simulated and experimented S_11_ and S_21_ plots. The proposed beam-steering phased-array has achieved an angular scanning range of 0° to 60° at 28 GHz, as shown in Figure 13g. The gain variation in the angular scanning range of ±60° is symmetrical with 15.6 dBi as peak gain. Two-dimensional (2D) radiation plots for various scanning angles of the proposed phased array are shown in Figure 13g.

**Figure 14:**
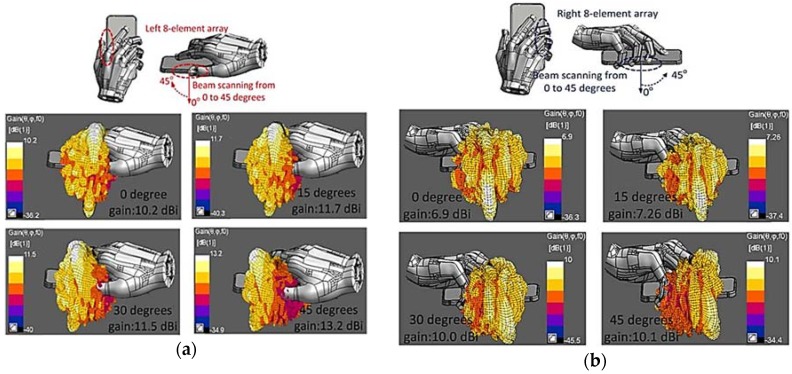
(**a**) Effects of user’s body on the proposed eight-element phased array with different beam-scanning angles positioned on the left side of mobile terminal. (**b**) User body effects on the eight-element phased array located along the right edge of mobile terminal at different beam-scanning angles. (Redrawn from [41])

Yu et al. also presented the effect of the user’s body on the gain and impedance matching of the proposed phased array, as illustrated in Figure 14a,b. The analysis of the user’s body effects on the proposed 16-element phased arrays shows that it can achieve a reasonable gain of at least 6.9 dBi, even with the user’s body effects.

Table 1 categorizes the performance of recent phased arrays for the 5G mobile terminal devices discussed in this section. A performance comparison has been made based on the beam-scanning capability, peak gain, and array elements of various phased arrays.

### 2.2. Millimeter-Wave Phased Arrays for 5G Access Terminals

In mm-wave cellular networks, backhaul systems and BSs will generally be deployed in crowdy environment on the poles, beacons, and building tops. The combined data to or from the multiple users will be transmitted with less delay from the mm-wave BS to the central hub through various mm-wave wireless or optical channels. The communication range for the outdoor mm-wave access link would be larger when compared to indoor mm-wave communication links, but the power consumption of the user end terminals cannot be increased. Because of the high signal attenuation at mm-wave frequencies, the effective communication distance of mm-wave systems is limited when compared to microwave signals. To reduce the increased path loss in outdoor environments, and owing to the user mobility, a suitable beamforming technique is required for implementation in cellular communication access links in the mm-wave frequency band. This can be realized by using the mm-wave BS equipped with high-gain phased arrays, where there is a relaxation of size and power consumption requirements. Phased arrays at millimeter-wave frequencies present a high-data-rate communication solution using high bandwidth and directional links between the BS and MS terminals.

#### 2.2.1. Millimeter-Wave PCB Phased Arrays

**Figure 15:**
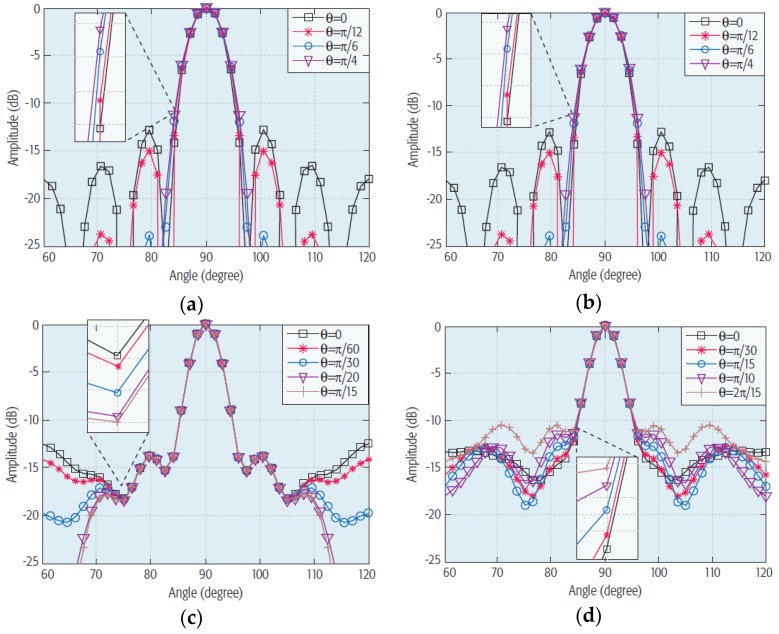
2D radiation plots of (**a**) 8 × 8 rectangular element array, (**b**) 16 cross-shaped element array, (**c**) 64 circular element array, and (**d**) 61 hexagonal element array (Redrawn from [9]).

In [9], Zhang et al. presented a comparative analysis of different antenna array architectures and beamforming techniques for outdoor mm-wave communication systems. Four different types of array architectures, i.e., an 8 × 8 rectangular element array, 64 circular element array, 61 hexagonal element array, and 16 cross-shaped element array architectures were analyzed, as depicted in Figure 15. The 2D radiation plots that were retrieved from 3D radiation patterns are illustrated in Figure 15. The analysis of different array architectures shows that the array architecture with circular elements has a larger coverage area with high gain and directivity compared to those of the other antenna array architectures.

**Figure 16:**
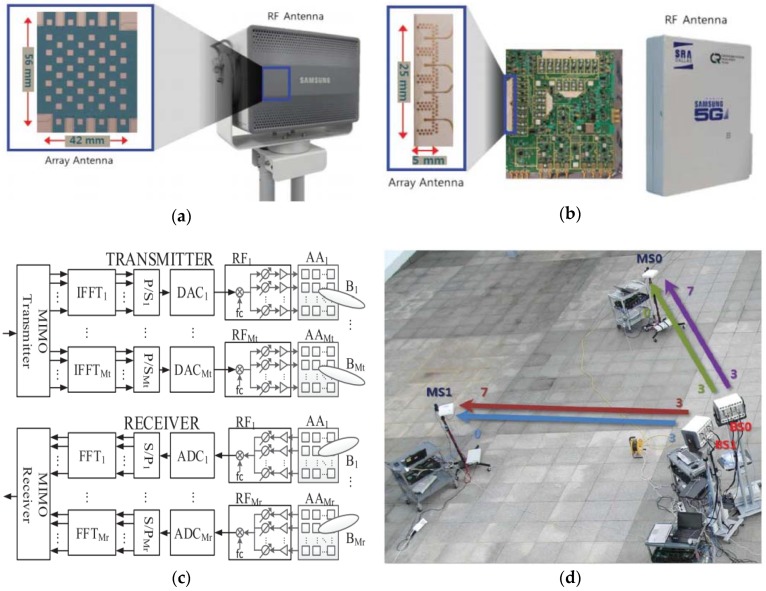
(**a**) Transceiver array antenna for BS. (**b**) Transceiver array antenna for MS. (**c**) Block diagram of testbed for mm-wave cellular communication with beamforming antenna array. (**d**) Testbed structure built at Samsung Electronics, Korea. (Redrawn from [13])

In [13], a testbed with a bandwidth of 800 MHz at an operating frequency of 28 GHz, which was built to test the practicality of mm-wave cellular communications at Samsung Electronics, (Suwon) Korea, was presented. The BS antenna array consisted of (6 × 8) 48 antenna elements that are grouped as subarrays in groups of three antenna elements to reduce the complexity of the system components. Each subarray provides a horizontal beam-scanning of 110° with the aid of a connected phase shifter, mixer, and an RF path. The MS antenna array was composed of two antenna subarrays, where each subarray provides horizontal beam-scanning of either 90° or 180°, with four elements designed on one of the sides of the RF board. The design overview of the BS and MS antenna arrays are shown in Figure 16a,b, respectively.

**Figure 17:**
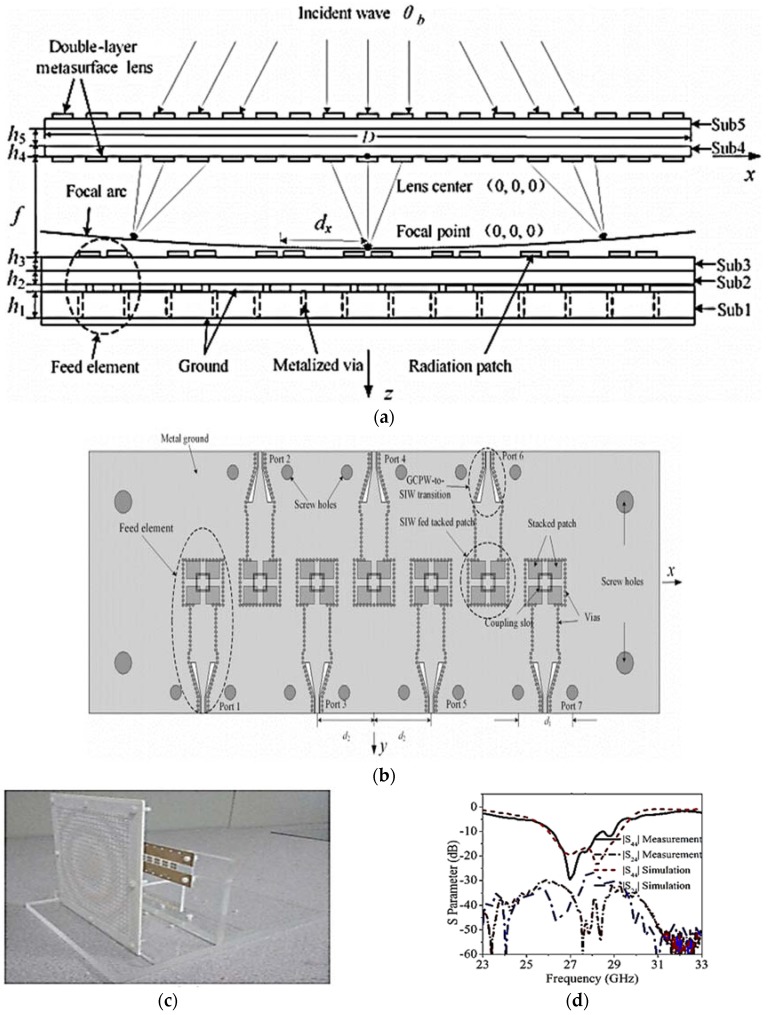

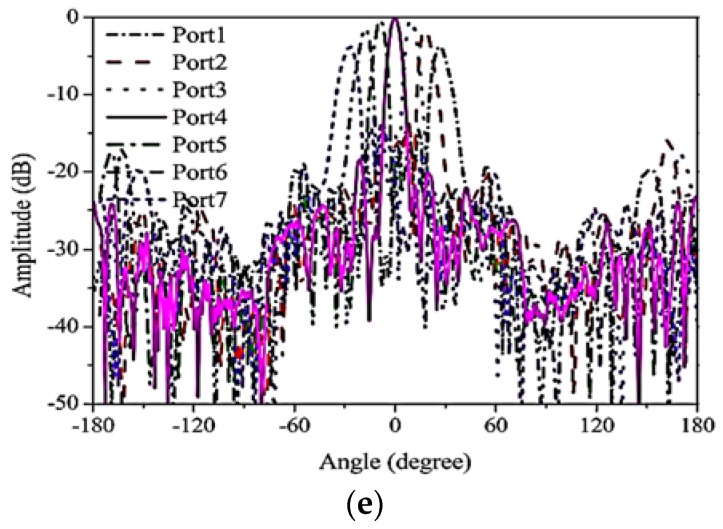
(**a**) Side view of seven-element antenna array combined with EM lens. (**b**) Top view of the seven-element SIW fed by a stacked patch antenna array. (**c**) Photograph of the lens integrated array antenna prototype. (**d**) Experimented and simulated 10-dB impedance bandwidth. (**e**) Measured scan beams of the proposed lens-fed phased-array antenna (Redrawn from [39]).

In [39], Jiang et al. proposed a novel array architecture for beamforming and multibeam massive MIMO systems using a metamaterial-based thin planar lens antenna. The proposed antenna comprises an EM lens combined with an antenna array. The EM lens together with an antenna array helps to increase the throughput and data rate of massive MIMO (m-MIMO). Both the lens and antenna elements were fabricated using PCB technology. Figure 17a,b respectively presents the side and top views of the proposed lens antenna. An array of a seven-element SIW-fed stacked patch antenna operating at 28 GHz is placed at the focal region behind the EM lens. Each subarray comprises four SIW-fed square patches as radiating patches. Each array element is connected to the input port using SIW-to-grounded-coplanar-waveguide (GCPW) transition feeding. The design overview of the seven-element SIW-fed stacked patch antenna array is given in Figure 17b. The proposed multilayer antenna array is fabricated on a Rogers RO4003C substrate having a dielectric constant of 3.55 and loss tangent of 0.0027. The thickness of the top and bottom layers is 0.508 mm and 0.813 mm, respectively. Both layers are joined together with a bonding layer of Rogers RO4450B, which has a dielectric constant of 3.54, loss tangent of 0.004, and height of 0.202 mm. A photograph of the fabricated prototype of the proposed seven-element array loaded with an EM lens is shown in Figure 17c. The measured 10-dB impedance matching ranges from 25.8–28.9 GHz, as shown in Figure 17d. The angular beam-scanning range of the proposed phased array is from −27° to +27°. Figure 17e shows the experimented radiation patterns of the proposed phased-array lens integrated antenna at 28 GHz. The measured peak gain of the lens-fed phased-array antenna is 24.2 dBi, with an efficiency of 24.5% at a frequency of 27.5 GHz.

**Figure 18:**
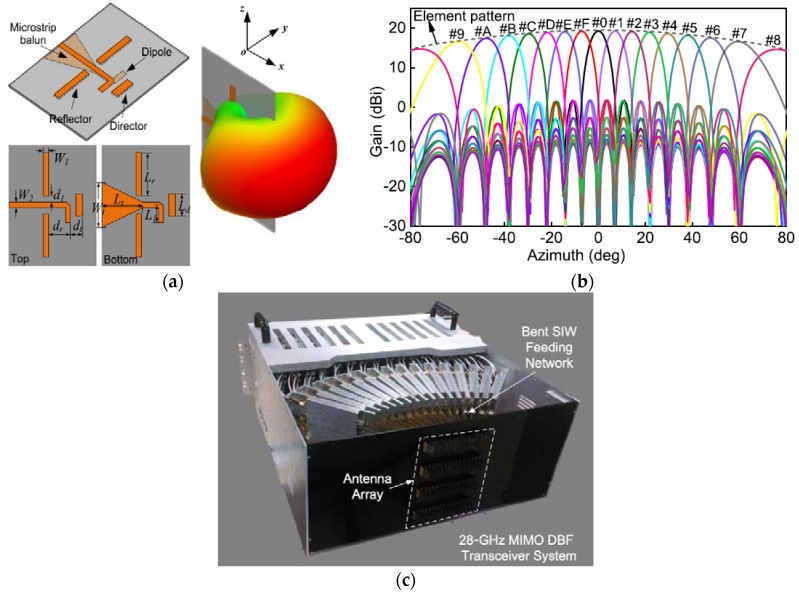
(**a**) Design overview of proposed Yagi-Uda antenna element with its 3D far-field radiation pattern. (**b**) Radiation patterns of 16-element DBF-based phased array in azimuth plane. (**c**) Photograph of the proposed DBF-based massive MIMO transceiver system with 64-antenna elements. (Redrawn from [15]).

In [15], mm-wave massive MIMO with digital beamforming (DBF) architecture operating at a 28-GHz frequency with an operating bandwidth of 500 MHz has been proposed. The proposed transceiver has a (16 × 4) 64-element antenna array configuration. A printed Yagi-Uda antenna element combined with a microstrip balun structure was chosen as the radiating element in the transceiver. Owing to the compact size, ease of fabrication, high gain, and low-cost factors, the proposed Yagi-Uda antenna element exhibits the best option to be considered for mm-wave MIMO transceivers [51]. Figure 18a depicts the design overview of the printed Yagi-Uda antenna element. The experimental reflection coefficient of the designed array is lower than −14 dB at a 28-GHz operating frequency with a bandwidth of more than 3 GHz. The wide azimuthal angular beam-scanning achieved by the proposed DBF-based phased-array system is ±67°, as shown in Figure 18b. The experimental Tx and Rx peak gains are 29 dBi and 27 dBi, respectively.

#### 2.2.2. Millimeter-Wave RFIC Phased Arrays

**Figure 19:**
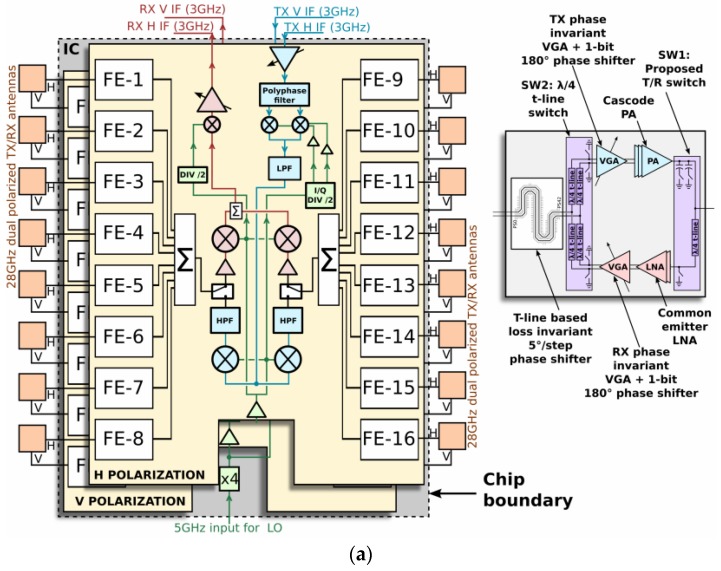

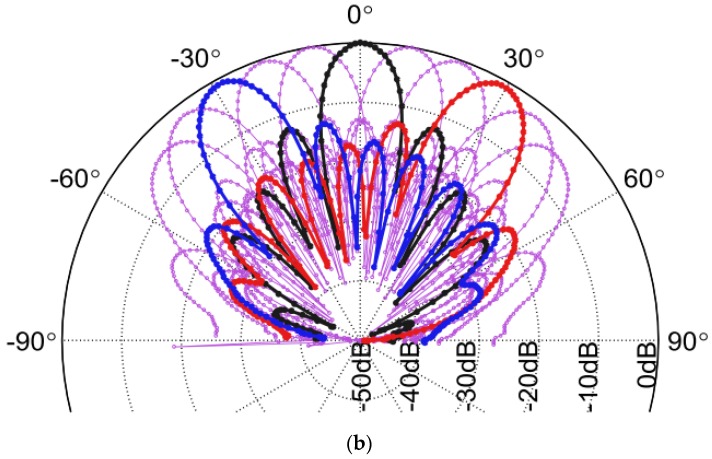
(**a**) RFIC architecture and schematic block diagram of 16-element phased array. (**b**) 64-element TX phased-array angular beam-scanning of ±50° (Redrawn from [53,65])

Along with the current IC-based packaging technology, there also exists an integrated analog-based phased-array solution proposed for 5G mm-wave communication [52,53,54,55,56,57,58,59,60,61,62,63,64,65,66,67,68,69]. In [53], a 32-element RFIC-based phased array operating at 28 GHz, which supports dual polarization and accurate beam-scanning capability with the advantage of high output power, was presented. The proposed RFIC reported in [53] is compact and efficient with respect to size, and scaled, which supports dual polarization at the Tx and Rx with in-packaged array technology. The proposed RFIC with a 32-transceiver (TRx) element phased array is implemented using a 0.13-µm-size SiGe BiCMOS (Bipolar Complementary Metal Oxide Semiconductor) process, as presented in Figure 19a. A packaged module of the proposed four ICs with 128 elements (32 each in four ICs) and 64 dual-polarized antenna elements providing two 64-element concurrent beams was proposed in [65] with a measured beam-scanning range of ±50° with a 1.4° resolution, as shown in Figure 19b.

**Figure 20:**
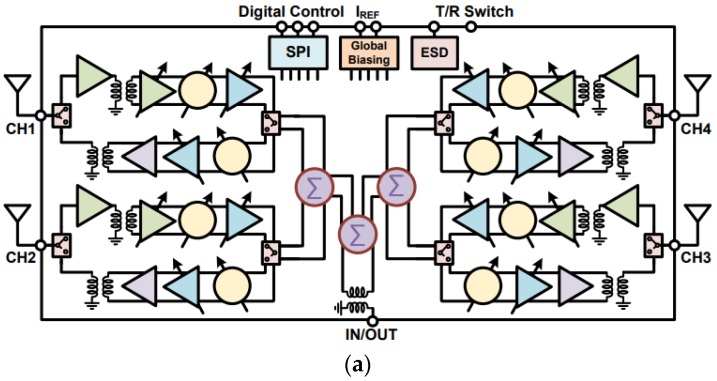

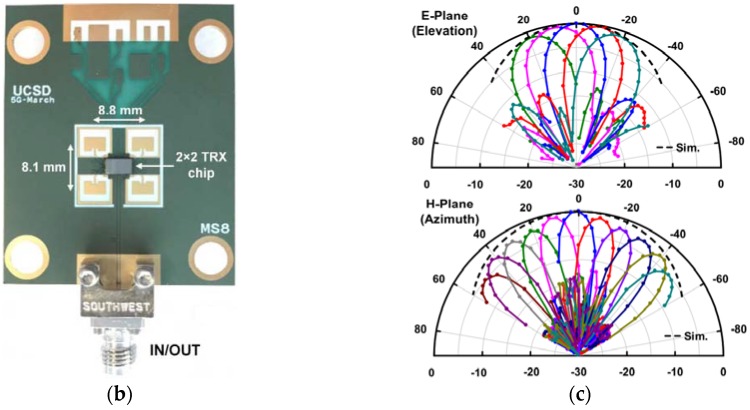
(**a**) Block diagram of 2 × 2 transmit/receive 5G phased array. (**b**) Photograph of the flip-chip test board with 2 × 2 transmit/receive 5G phased array. (**c**) Measured E and H-plane patterns of 4 × 8 transmit/receive 5G phased array in Rx mode (Redrawn from [54,60]).

In [54], an IC-based transceiver with a flip-chip package phased array operating at 28–32 GHz was proposed for 5G mm-wave communication. The proposed IC chip comprised four separate TRx channels along with 6-bit phase shifters and a 4-bit 14-dB gain control. Figure 20a shows the block diagram of 2 × 2 TRx 5G mm-wave phased arrays. The proposed antenna design shown in Figure 16b has a 10-dB reflection coefficient of 0.5 GHz. The peak array gain is 18 dBi and 12 dBi for the Tx and Rx ends, respectively. The measured EIRP (Effective Isotropic Radiated Power) at 29 GHz is 24.5 dBm. The photograph of the flip-chip test board with 2 × 2 antenna elements is shown in Figure 20b. Based on the proposed beamformer packaged module, a 32-element (4 × 8) TRX array with PCB integrated microstrip antennas on a four-layer PCB stack was proposed in [60]. The angular beam-scanning of ±50° in the azimuth and ±25° in the elevation plane was achieved, as shown in Figure 20c.

**Figure 21:**
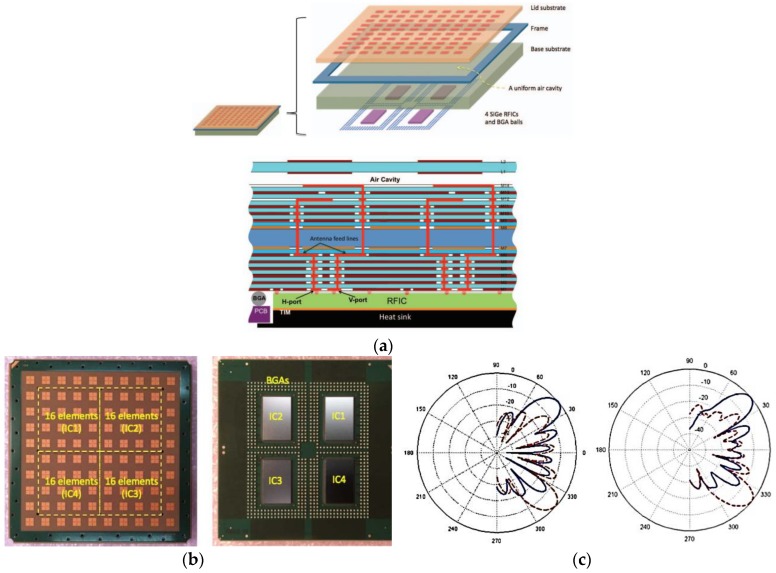
(**a**) Overview of antenna array assembly with transceiver ICs. (**b**) Photograph of the assembled antenna array package with four transceiver ICs. (**c**) 64-element TX phased-array angular beam-scanning of ±40° (Redrawn from [67]).

In [67], a multilayer 64-element dual-polarized antenna-in-package assembly with four-SiGe BiCMOS multi-chip phased array operating at 28 GHz was proposed for 5G mm-wave communication. The proposed module has four mounted SiGe BiCMOS transceiver ICs supporting dual polarization in Tx and Rx modes. The proposed antenna array has a 3-GHz bandwidth at an operating frequency of 28 GHz. The measured Tx gain is around 35 dBi for the 64-element antenna array. The angular beam-scanning of ±40° in the azimuth and elevation plane was achieved, as shown in Figure 21c.

Table 2 shows the performance of recently developed phased arrays for 5G access terminals demonstrated in this section. A performance comparison was made based on the beam-scanning capability and array elements of various phased arrays.

## 3. Discussion

Although mm-wave technology is being considered overall for future 5G wireless communication systems, there remains the need for improvement and changes in current mm-wave architectures and the expected commercial designs to be used in mm-wave cellular communication networks [4,6,9]. A few basic improvements are required with respect to the deployment and implementation of mm-wave cellular networks. Sophisticated antenna array designs are proposed to improve the performance of wireless communication systems in terms of high gain and coverage area. Because there is a trade-off between the gain and bandwidth, phased arrays structures offer an increased angular beam-scanning range along with high gain [70]. Various types of phased-array structures have been developed in the last few years. For the BS array antenna, there remains a relaxation of the space factor for designing complicated phased-array designs with increased spatial beam-scanning. However, in the case of MS antennas, it is more demanding to implement phased arrays in cellular handsets because of the limited space, user mobility, and user effects. Therefore, in this article, we discussed in detail the design constraints and implementation challenges of mm-wave phased arrays for 5G mobile terminals (MSs) and access terminals (BSs).

To obtain the directional fan-beam radiation patterns in both the vertical and horizontal directions, two separate phased arrays positioned on the top and bottom of the chassis of a cellular handset were demonstrated in [10]. In [49], 3D beam-scanning was achieved by employing a folded 3D structure using three subarrays at the top position, but there is a limitation in terms of the implementation of the 3D array structure in the cellular handset. To address this limitation, 3D coverage was achieved in [50] using the surface waves of three identical slotted subarrays by switching the main beam direction to separate regions. Each subarray behaves similar to a phased array to tilt the beam to a separate region.

To address the polarization mismatch [7,46] and incurred losses owing to the mobility of the user and various kinds of motions experienced by the mobile terminals at different angles, multi-polarized, i.e., horizontally polarized and vertically polarized antennas are combined in a subarray to achieve the diversity gain, while enhancing the efficiency of the transmission and reception at mm-wave frequencies [47]. To achieve polarization diversity along with the enhanced bandwidth, printed horizontally polarized and vertically polarized quasi-Yagi-Uda antennas were implemented [51]. Enhanced beam-scanning is achieved by combining horizontally polarized and vertically polarized antennas in an array using the side-by-side placement of these subarrays in a cellular handset [44,47].

Because of the constraints of space and position in cellular handsets, much research has been performed on the structure of mm-wave phased arrays with different handset positions [28,37,41,50]. Most of the phased arrays for 5G mobile terminals are single-layer substrate planar phased arrays to better fit into the chassis of cellular phones [42,48,49,50]. Further research efforts have been attempted on multilayer mesh-grid phased arrays [7] and SIW phased arrays [37].

Although most research has been performed on low-cost substrate boards [71,72], such as PCB-based phased arrays, there is little compliance with the metallic frame casing of modern cellular devices [56,57]. However, the study in [41] introduced a set of two eight-element cavity-backed slotted subarrays on both sides of the metallic casing of a cellular device. The study in [40] illustrates the degree to which the human body is vulnerable to electromagnetic fields (EMF) owing to the designed phased-array antennas in terms of the specific absorption rate (SAR). The authors in [40] successfully reduced the SAR to 0.88 W/kg by using slotted subarrays on the top and back of the metallic casing of a cellular handset.

To address the increased path loss at mm-wave frequencies and the lower power consumption compared to that of sub-6-GHz communication links, LOS communication using phased-array techniques with an array antenna gain is required. Various antenna array architecture designs for outdoor mm-wave communication with different beamforming techniques, i.e., analog, digital, and hybrid techniques, were analyzed [9]. The antenna array design with circular elements is more robust to vibrations and is therefore the best candidate for outdoor mm-wave communication in terms of beam gain, gain fluctuation, and beam misalignment of phased-array antennas. Analog beamforming compensates for the high path loss by generating high beamforming gains, whereas digital beamforming presents a better performance with an enhanced structural complexity. Therefore, a combination of analog and digital beamforming array architectures on both the transmitter and receiver ends has become the focus of research in recent years days based on the trade-off between the flexibility and performance in outdoor mm-wave communications [55,73].

Cost is an important factor that needs to be significantly lowered owing to the widespread use of phased arrays in 5G wireless communication. Therefore, ICs that are based on silicon technology (SiGe or BiCMOS) [34,35,54,58,59] along with low-cost PCB technology [35,36,60] should be considered. Scalable, RFIC-based phased arrays that are capable of beam-scanning in both the horizontal and vertical planes currently attract much attention from researchers [60].

## 4. Conclusions

In this review, we demonstrated recently proposed phased arrays for 5G mobile terminals and access terminals. These state-of-the-art phased arrays are compact in size, low-cost, and have enough beam-scanning and coverage range. The effects of the user’s body and human exposure to EM waves by these state-of-the-art phased arrays with 3D coverage designed on single-layer and multilayer substrates were illustrated. In addition, multi-polarized antenna arrays and the space constraints of mobile terminals were presented and discussed. Various beamforming techniques using state-of-the-art mm-wave phased-array architectures to mitigate the co-channel interference and to overcome the path loss were proposed.

## Figures and Tables

**Table 1:** Performance variation of various phased arrays for 5G mobile terminals.

Ref #	No. of Subarrays	Elements Per Subarray	f_o_ (GHz)	BW (GHz)	Beam-Scanning (deg)	Peak Gain (dBi)	Substrate	Array Position
[10]	2	1 × 16	27.9	=1	±70°	10.5	Multilayer-PCB	Top and Bottom
[46]	4	1 × 16	28	-	±80°	-	Multilayer-FR4	Four Corners
[49]	3	1 × 8	21.5	=1	±90°	12.5	Nelco N9000	Top
[48]	1	1 × 8	28	>1	±70°	12	N900 PTFE	Top
[50]	3	1 × 8	28	=2	±73°, ±128°, ±20°	12.5	Nelco N9000	Top
[41]	2	1 × 8	28	>2.5	±60°	15.6	Metallic casing	Left and Right
[42]	1	1 × 8	28	>8	±90°	12	Taconic RF-30	Top
[47]	3	1, 1 × 4, 1	36	>2	-	11	Multilayer-PCB	Top (Mid + Corners)

**Table 2:** Performance variation of various phased arrays for 5G access terminals.

Ref #	Array/IC Elements	Elements in Package	TX/RX Polarizations Per IC	f_o_ (GHz)	Beam-Scanning (deg)	Gain (Tx/Rx) (dBi)	Technology	TX/RX Interface	Package Specification / Integration Level
[9]	32 × 32	1024	-	28	-	-	Single-layer PCB	-	**(HUST)** Array Antenna architecture
[13]	6 × 8	48	-	28	±110°	21	Single-layer PCB	-	**(Samsung)** MIMO Antenna transceiver
[39]	1 × 7	7	-	28	±27°	24.2	Multilayer PCB	-	Antenna only
[15]	16 × 4	64	-	28	±67°	29/27	Single-layer PCB	-	**(Bell Labs)** MIMO transceiver with DBF architecture
[55]	4 × 8	32	-	27.925	±30°	18	Single-layer PCB	-	**(Samsung)** Array Antenna architecture
[53,65]	16 × 2	128	2/2	28	±50°	32/34	0.13 µm SiGe BiCMOS	3 GHz IF	**(IBM)** Antenna in package
[54,60]	2 × 2	4	1/1	29	±50°, ±25°	18/12	0.18 µm SiGe BiCMOS	RF	**(UCSD)** Antenna on PCB
[61]	2 × 4	8	1/1	28	±20°	14	28 nm CMOS	Analog IQ BB	**(LG)** Antenna in package
[63]	4 × 6	24	2/2	28	±45°	44/34	28 nm CMOS	6.5 GHz IF	**(Qualcomm)** Antenna in package
[66]	2 × 2	4	-	28	±45°	-	Multilayer PCB and Flip-chip module	-	**(Qualcomm)** Antenna on PCB with packaged ICs
[67]	4 × 4	64	2/2	28	±40°	35	Multilayer PCB + 4 SiGe BiCMOS	3 GHz IF interface	**(IBM)** Antenna in package
